# Predominance of t355/ST152/SCCmec V clonal type among PVL-positive MRSA isolates in a tertiary care hospital in Belgrade, Serbia

**DOI:** 10.1371/journal.pone.0273474

**Published:** 2022-09-08

**Authors:** Bojan Rakonjac, Zorica Lepšanović, Vesna Šuljagić, Branko Jovčić, Milan Kojić, Anders Rhod Larsen, Momčilo Đurić, Ivana Ćirković

**Affiliations:** 1 Military Medical Academy, Belgrade, Serbia; 2 Medical Faculty, University of Defence, Belgrade, Serbia; 3 Institute of Molecular Genetics and Genetic Engineering, Univerity of Belgrade, Belgrade, Serbia; 4 Department of Microbiological Surveillance and Research, Statens Serum Institut, Copenhagen, Denmark; 5 University of Belgrade-Faculty of Medicine, Institute of Microbiology and Immunology, Belgrade, Serbia; Tribhuvan University, NEPAL

## Abstract

Epidemiology of methicillin-resistant *Staphylococcus aureus* (MRSA) is continually changing. Frequency of genotypes typical for community-associated MRSA (CA-MRSA) is increasing in hospitals, as well as resistance to antimicrobial agents. Moreover, different clones predominate in different geographic regions, and temporal shifts occur in the predominant clonal type. The aim of this study was to estimate the prevalence of MRSA, CA-MRSA and PVL-positive MRSA isolates from patients hospitalised in the Military Medical Academy (MMA) and from outpatients, and to perform genotyping of PVL-positive MRSA isolates. MRSA isolates were obtained by standard microbiological techniques. PVL-positive MRSA were detected by single PCR. Determination of SCC*mec* types in MRSA isolates was done using multiplex PCR and genotyping of PVL-positive MRSA by PFGE, MLST and *spa* typing. The prevalence of MRSA among *S*. *aureus* isolates from different clinical specimens was 43.4%. In outpatients the prevalence of MRSA was 3.2%. SCC*mec* types specific for CA-MRSA were found in 26% of MRSA isolates from hospitalised patients. In groups, hospitalised patients and outpatients, the prevalence of PVL-positive MRSA isolates was 4%, and all of them harboured SCC*mec* type V genetic element. PFGE revealed minor differences between four groups of PVL-positive MRSA isolates, but all of them belonged to ST152, and all except one were of the *t*355 *spa* type. High prevalence of MRSA and CA-MRSA in MMA, especially the presence of PVL-positive CA-MRSA, represent a serious health threat for patients. Genotype t355/ST152/SCC*mec* V is the dominant MRSA clone among PVL-positive CA-MRSA.

## Introduction

Methicillin-resistant *Staphylococcus aureus* (MRSA) is still one of the most common human pathogens that cause healthcare-associated infections worldwide. Although its prevalence has been decreasing, community-associated MRSA (CA-MRSA) is responsible for a new wave of emergence that appeared in 1990s, outside healthcare units [1]. This pathogen, in contrast to healthcare-associated MRSA (HA-MRSA), has caused infections in previously healthy young individuals with no history of healthcare contact, and is more susceptible to antibiotics and more virulent [2]. CA-MRSA expresses Panton-Valentine leukocidin (PVL) and other genetic elements that enhance its virulence [3]. PVL is a bi-component toxin encoded by the *lukF-PV* and *lukS-PV* genes carried on a lysogenic bacteriophage, with the ability to lyse leukocytes. Production of PVL is connected with severe MRSA skin and soft tissue infections such as recurrent furunculosis, necrotising pneumonia and fasciitis [4].

General genetic difference between CA-MRSA and HA-MRSA is in Staphylococcal Cassette Chromosome *mec* (SCC*mec*) element. This genetic element is smaller and usually of types IV or V in CA-MRSA strains. When CA-MRSA spread to hospitals, this pathogen acquired genes for resistance under selective pressure of antibiotics, and its resistance pattern resembled that of HA-MRSA. Typing of SCC*mec* element and the presence of PVL genes, beside multilocus sequence typing (MLST) and sequencing of the repeat regions of *S*. *aureus* protein A (*spa* typing), are currently the only ways to distinguish CA- from HA-MRSA beside whole genome sequesting [2].

The aim of the study was to estimate the prevalence of MRSA, CA-MRSA and PVL-positive MRSA isolates from patients hospitalised in the Military Medical Academy (MMA) and from outpatients, and to identify the clonal types of PVL-positive MRSA.

## Material and methods

### Study design, site and population

A cross-sectional study was conducted at the Military Medical Academy (MMA), a teaching 1000-bed tertiary-care hospital of the University of Defence in Belgrade, Serbia. MMA acts as one of the major referral health facilities in Serbia with 35,000 patients hospitalised, 20,000 surgical procedures performed, more than half a million specialist examinations and around 3 million diagnostic and laboratory procedures carried out each year.

Clinical samples originated from patients hospitalised at different clinical departments, with the majority being from the surgical Intensive Care Unit (ICU), neurosurgery, general surgery and internal ICU were collected between June 2015 and October 2016.

Outpatient screening samples originated from patients that meet the criteria for CA-MRSA strains [1, 3] were collected upon hospital admission between June 2015 and May 2017.

Participation in this study was voluntary and all participants signed the written informed consent form prior to their inclusion in the study. Descriptive information regarding participants’ age, gender, diagnosis, department and period of hospitalisation was collected. The study protocol was approved by the Ethics Committee of Medical Faculty, University of Defence, Belgrade, Serbia [number: 18042013].

### Specimen types, culture method and identification

Clinical strains analysed in the study were isolated from different specimens: aspirates, wound swabs, blood, cerebrospinal fluid, sputum and peritoneal fluid. Samples from outpatients included two swabs, one taken from both anterior nares and one from the throat.

After the collection, all specimens were processed within 2h. *S*. *aureus* isolates were obtained by standard microbiological techniques. Specimens were cultured on Columbia agar with 5% sheep blood (bioMérieux, France), incubated for 24 hours aerobically at 37°C, and identified by the tube coagulase test with rabbit plasma (Torlak, Belgrade) and confirmed by MALDI-TOF MS (VITEK MS, bioMérieux) [5, 6]. Methicillin resistance in *S*. *aureus* strains was determined by the disk diffusion method using cefoxitin disk in accordance with the European Committee on Antimicrobial Susceptibility Testing (EUCAST) recommendation (http://www.eucast.org).

MRSA isolates were stored in dextrose broth at -20°C, and re-cultivated on Columbia agar with 5% sheep blood (bioMérieux) for further examination.

The molecular characterisation of MRSA isolates was performed analysing 100 random MRSA clinical isolates, and all of 50 MRSA outpatient isolates.

### Antimicrobial susceptibility testing

Susceptibility to penicillin, cefoxitin, erythromycin, clindamycin, fusidic acid, gentamicin, ciprofloxacin, levofloxacin, rifampin, tetracycline and trimethoprim/sulfamethoxazole (BioRad, USA) was determined by the disk diffusion method and to vancomycin and teicoplanin by gradient E test (bioMérieux) in accordance with the EUCAST recommendation (http://www.eucast.org).

### PCR amplification and SCC*mec* typing

For PCR amplification, bacterial DNA was prepared using a kit for DNA purification (GeneJET Genomic DNA Purification Kit, Fermentas, Thermo Fisher Scientific, EU). The identity of MRSA isolates was confirmed by PCR for *nuc* and *mec*A genes [6, 7]. The primers used to amplify a 433 bp region of *luk-*PV genes and PCR conditions were previously described by Darboe et al. [8]. Typing of SCC*mec* genetic element was performed as multiplex PCR amplification using the primers and PCR conditions described by Milheiriço et al. [9]. Referent strains were *S*. *aureus* ATCC 43300 and *S*. *aureus* ATCC 25923 (Microbiologics, Inc. St Cloud, MN, USA).

### Pulsed field gel electrophoresis (PFGE)

Pulsed field gel electrophoresis (PFGE) analysis of native, *SpeI*-digested genomic DNA from *luk-*PV-positive MRSA isolates was carried out using a contour-clamped homogeneous electric field system (2015 Pulsafor unit; LKB Pharmacia, Sweden) in 0.59 TBE (45 mmol/l Tris base, 45 mmol/l boric acid, and 1 mmol/l EDTA pH 8). The interpretation of results was performed as described previously [1, 10].

### Multilocus sequence typing (MLST) and *spa* typing

The total DNA isolated from the representative *luk-*PV-positive MRSA strains was subjected to MLST analysis. Sequencing of the internal fragments of the seven house-keeping genes was performed as described at https://pubmlst.org/saureus/info/primers.shtml. Representatives of *luk-*PV-positive MRSA strains were subjected to *spa* typing as previously described [11].

## Results

*S*. *aureus* strains were obtained during routine diagnostic testing at the Department of Microbiology, MMA. *S*. *aureus* was isolated from 569 different clinical specimens, and 247 (43.4%) of them were MRSA. In the period of outpatients’ specimen collection, *S*. *aureus* was detected in 1565 of them. Methicillin resistance was confirmed in 50 *S*. *aureus* isolates (3.2%).

**Fig 1:**
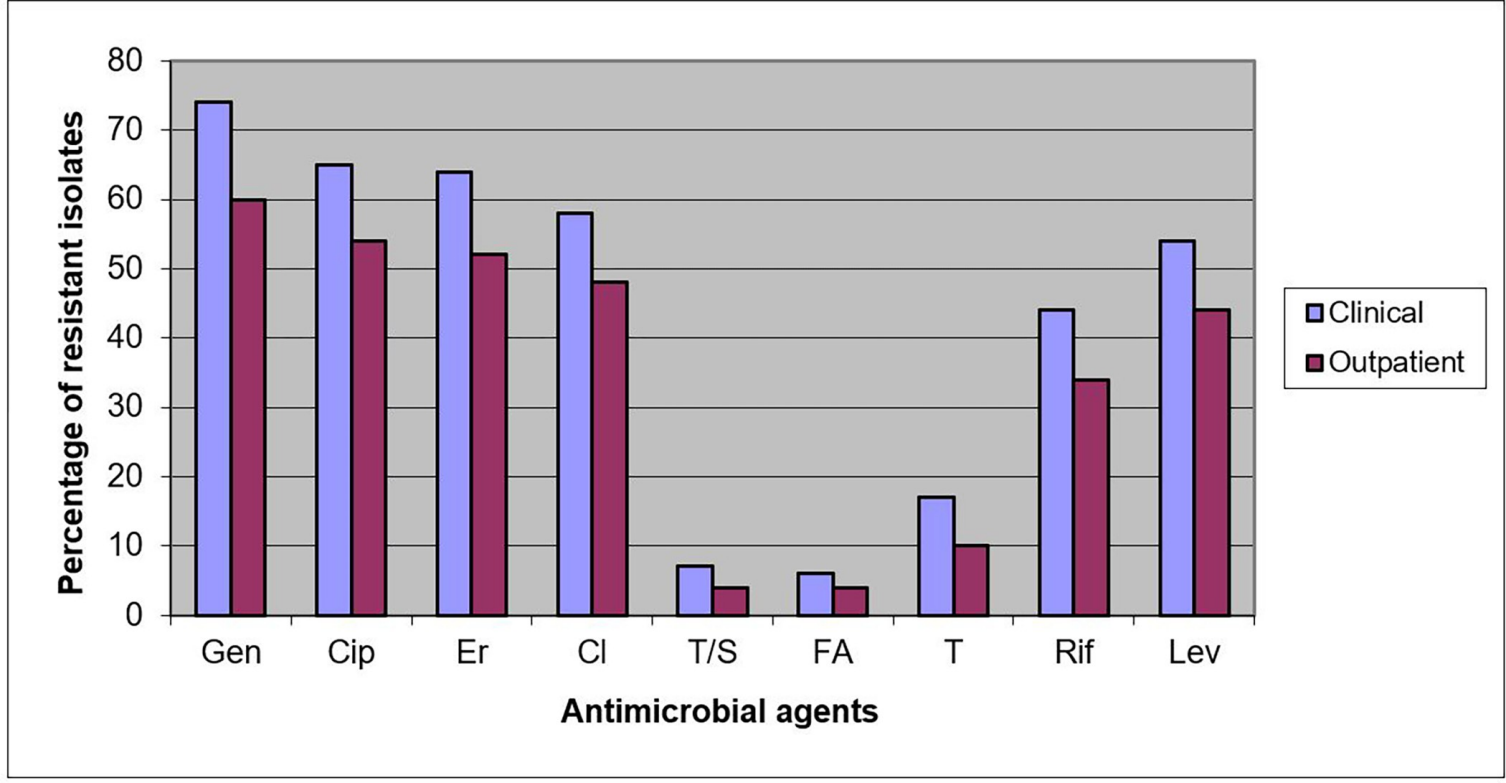
Antimicrobial resistance in clinical and outpatient MRSA isolates. Gen-gentamicin; Cip-ciprofloxacin; Er- erythromycin; Cl—clindamycin; T/S trimethoprim/sulfamethoxazole; FA -fusidic acid; T—tetracycline; Rif—rifampicin; Lev- levofloxacin.

**Fig 2:**
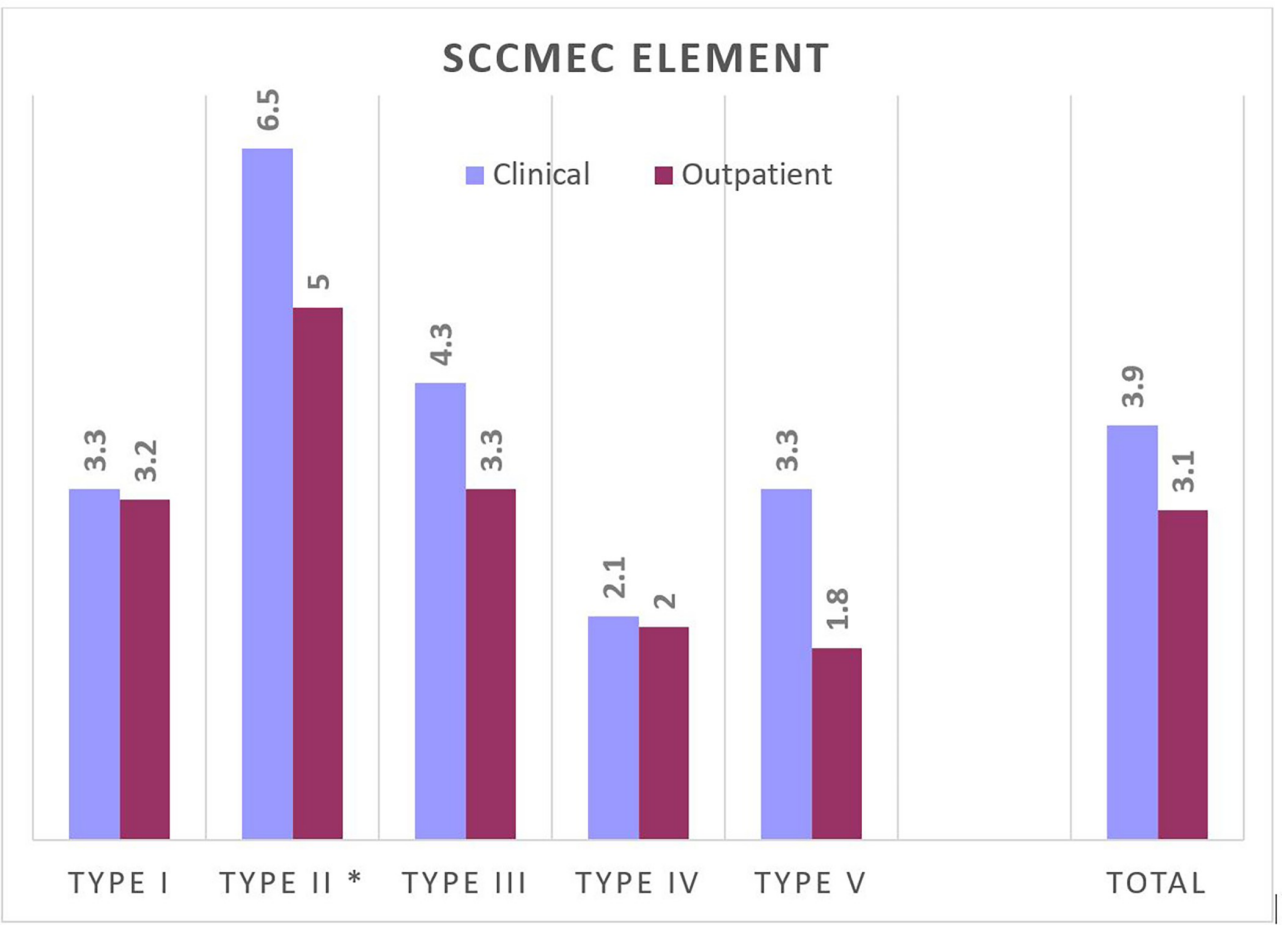
Average antimicrobial resistance of clinical and outpatients MRSA isolates with different SCC*mec* types. *A small number of isolates.

Apart from penicillin and cefoxitin, MRSA isolates (86% of clinical isolates, and 68% of outpatient isolates) harboured resistance to non-β-lactam antimicrobial agents. All clinical and outpatient isolates were susceptible to vancomycin and teicoplanin. The largest number of clinical isolates was resistant to gentamicin (74%), ciprofloxacin (65%), erythromycin (64%), clindamycin (58%) with 18 strains exhibiting inducible resistance, levofloxacin (54%) and rifampin (44%) (Fig 1). Similarly, MRSA outpatient isolates were resistant to gentamicin (60%), ciprofloxacin (54%), erythromycin (52%), clindamycin (48%), with 5 strains exhibiting inducible resistance, levofloxacin (44%) and rifampin (34%). MRSA isolates of clinical and outpatient origin were on average resistant to 3.9 and 3.1 non-β-lactam antimicrobial agents, respectively (Fig 2). In MRSA isolates with SCC*mec* type I and with SCC*mec* type IV, the average resistance was almost the same, regardless of the origin of the isolates.

SCC*mec* typing revealed that isolates with type III genetic element were the most abundant (64%) in clinical MRSA isolates (Table 1). Other typical HA-MRSA types were scarcely found: type I in 7%, and type II in 2% of MRSA isolates. SCC*mec* types IV and V, typical for CA-MRSA, were detected in 15% and 11% of clinical isolates, respectively. One MRSA isolate could not be typed using this method.

**Table 1:** SCC*mec* types of MRSA isolates from clinical and outpatientsʹ samples.

Source of strains	SCC*mec* types								Total
	n (%)								
	HA-MRSA types				CA-MRSA types				
	I	II	III	Total	IV	V	Total	Non-typeble	
				I-III			IV-V		
Clinical samples	7	2	64	73	15	11	26	1	100
	(7)	(2)	(64)	(73)	(15)	(11)	(26)	(1)	(100)
Outpatients	9	2	6	17	16	12	28	5	50
	(18)	(4)	(12)	(34)	(32)	(24)	(56)	(10)	(100)

In outpatient MRSA isolates, typing results were as follows: type I SCC*mec* element was found in 9 (18%) isolates, type II in 2 (4%), and type III in 6 (12%) isolates. SCC*mec* types typical for CA-MRSA were more frequent: type IV was found in 16 (32%) and type V in 12 (24%) isolates. In five MRSA isolates the type of SCC*mec* element could not be determined.

**Fig 3:**
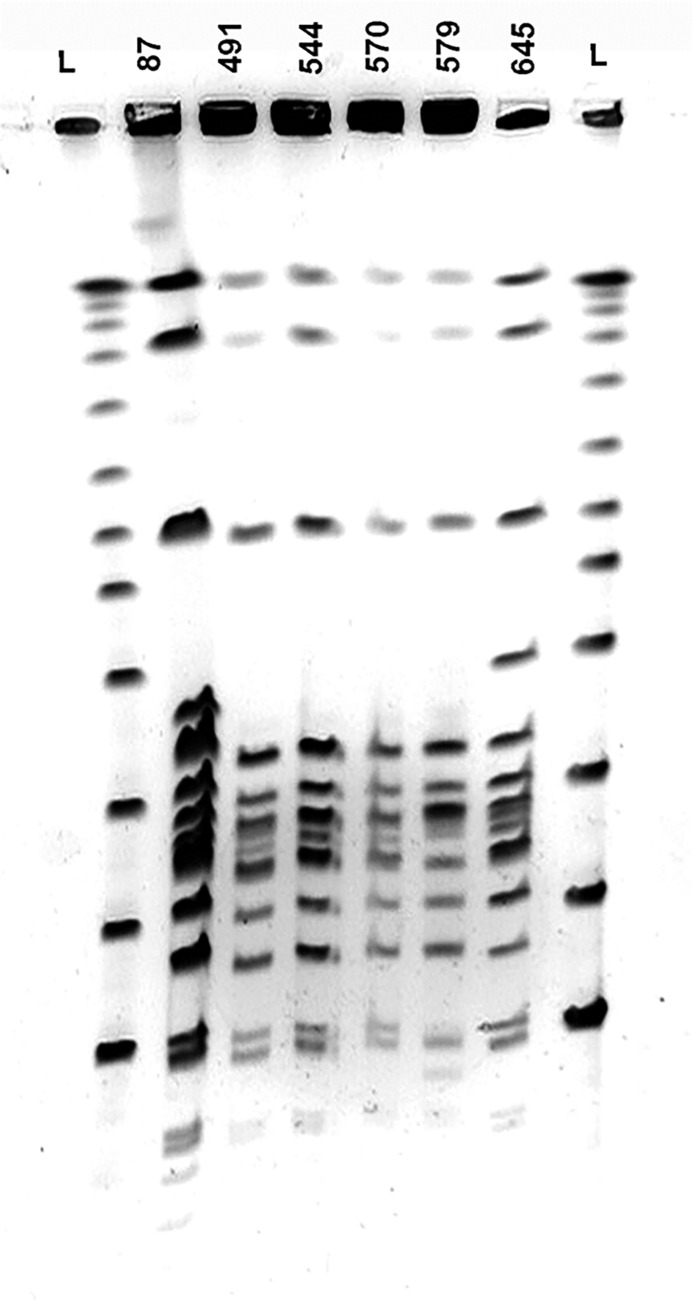
*Sma*I-PFGE macrorestriction profile of *luk-*PV*-*positive SCC*mec type* V MRSA isolates. L, λ concatemers (New England Biolabs); MRSA isolates: 87, 491, 544, 570, 579, 645.

All MRSA isolates were screened for the presence of genes encoding PVL. Specific amplified fragment corresponding to *luk-*PV genes was detected in four (4%) clinical isolates and two (4%) outpatient isolates. All six isolates harboured SCC*mec* type V element. PFGE results of *luk-*PV-positive isolates revealed four different pulsotypes: isolates 87, 579 and 645 were all different from one another, and the fourth group consisted of isolates 491, 544 and 570 with identical profile (Fig 3). PVL-positive MRSA isolates were further subjected to MLST analysis and *spa* typing. The profile of analysed alleles was the same in all strains: *aroE–* 75, *pta–* 13, *glpF–* 49, *arcC*– 46, *gmk*– 44, *tpi*– 68, *yqil*– 60. This allele combination corresponded to ST152 sequence type. Regardless of some differences in PFGE macrorestriction profiles of six MRSA strains, all of them belonged to the same sequence type, ST152, already known as a toxin producer. *Spa* type *t*1096 was found in one strain of outpatient origin (Table 2). All other tested strains, one from outpatient and four from clinical samples, were of *spa* type *t*355.

**Table 2:** Molecular characterisation of PVL-positive MRSA isolates.

Strain (source)	SCC*mec* type	PFGE type	MLST	*spa* type
**87**	V	A	152	*t*1096
**outpatient**				
**491**	V	B	152	*t*355
**outpatient**				
**544**	V	B	152	*t*355
**clinical**				
**570**	V	B	152	*t*355
**clinical**				
**579**	V	C	152	*t*355
**clinical**				
**645**	V	D	152	*t*355
**clinical**				

## Discussion

The prevalence of MRSA varies in different regions of the world. At the beginning of this century, the lowest prevalence of MRSA in hospitals and other healthcare settings in Europe was in northern countries and the Netherlands, 0.6%, while it went up to 44.7% in other European countries [12]. Non-European countries with the highest MRSA prevalence in hospitals were Japan (34.9%), Latin America (40.4%), and Australia (66.8%) [12]. In US hospitals the prevalence of MRSA was stable or has declined [13–15]. After that period, MRSA incidence has been decreasing in Europe [16]. However, according to the latest available data in Europe, the lowest prevalence of MRSA in hospitals and other healthcare settings from blood samples and cerebrospinal fluid (CSF) was in northern countries: Norway (1.1%), Sweden (1.8%), Denmark (2.2%), in the Netherlands (1.6%), while in other European countries it was up to (46.7%) in Romania or (45%) in the North Macedonia. (www.ecdc.europa.eu/sites/default/files/documents/Country%20summaries-AER-EARS-Net%20202019.pdf). The latest available data from Serbia shows the value of 26% (Central-Asian-and-European-Surveillance-of-Antimicrobial-Resistance.-Annual-report-2020-eng.pdf).

In this study, the prevalence of HA-MRSA among *S*. *aureus* isolates at the MMA hospital is 43.4%, which is higher than the prevalence data in the Serbia. The reasons for this are manifold: MMA is one of the largest tertiary health care hospitals in the Serbia with 1000 patient beds. Our patients gravitate from all parts of the country. MMA is not part of the surveillance network that comprises 24 laboratories (37% being tertiary care hospitals and 63% secondary care hospitals) in Serbia. Furthermore, MRSA hospitals strains in our study had previously been isolated from sterile sites such as CSF, blood and pleural puncture, which corresponds to the national data but we also included a number of clinically significant samples in our study that do not basically belong to sterile regions (swabs of various wounds, skin infections, tissue samples, bronchial aspirate, sputum). In a similar study in another part of Serbia, the prevalence of MRSA infections was 45.8% [17].

In our study the prevalence of CA-MRSA carriage was 3.2%, slightly lower than the prevalence found in Pomoravlje Region of Serbia (3.8%) in 2016 [17]. Reported MRSA prevalence in the community is low worldwide, but with an increasing tendency [12]. According to the results from nine European countries, the prevalence of MRSA in healthy people varies from 0% in Sweden to 2.1% in Belgium [18]. In the Asia-Pacific region the prevalence of CA-MRSA among the general public ranged from 0.3% to 23.5% [19].

The detection of CA-MRSA genotypes in hospitals is always a cause for concern because infections caused by them are usually severe, hospitalisation prolonged and mortality rates high [20]. In our study, the ratio of SCC*mec* types typical for HA-MRSA and SCC*mec* types typical for CA-MRSA was 73% vs. 26% in clinical isolates, while in outpatients the ratio was 34% vs. 56%. Both ratios are reflecting the changing epidemiology of CA-MRSA. When CA-MRSA entered the hospitals, as a consequence, the exchange of genetic determinants encoding resistance and virulence occurred between CA- and HA-MRSA strains. Besides, this exchange can occur in community where HA-MRSA strains can be transferred from hospitals, but less extfrequently. Although the average number of resistance determinants in our study was higher in MRSA isolates from hospitalised patients than from outpatients (3.5 vs. 2.2 antibiotics), in MRSA with SCC*mec* type I and MRSA with SCC*mec* type IV the average number of resistance determinants is almost the same in both groups of isolates, hospitalised patients and outpatients.

All six PVL-positive MRSA isolates in our study, regardless of the source, carried SCC*mec* type V genetic element, and all belonged to ST152 genotype. Four PVL-positive ST152/SCC*mec* type V clinical isolates were not epidemiologically related and were with different resistant patterns. This result may suggest that PVL-positive ST152/SCC*mec* type V strains have not circulated in hospitals, specifically at MMA for years. PFGE genotyping of all PVL-positive isolates revealed four patterns, although with minor differences that could be due to the presence of different mobile genetic elements in strains, since the pattern of the main fragments is almost identical. PVL-positive ST152/SCC*mec* type V have been identified in Serbia since 2008 when a national surveillance study revealed the presence of four PVL-positives out of 162 MRSA clinical isolates (2.5%) [21]. Three of four PVL-positives MRSA isolates in that study belonged to ST152/SCC*mec* type V, each with a different *spa* type: *t*6371, *t*1183, and *t*355. The remaining PVL-positive MRSA isolate was typed as *t*044/ST80/SCC*mec* type IV. In another study of nasal MRSA carriage among medical students in Belgrade, two PVL-positive MRSA strains were detected: one was characterised as *t*044/ST80/SCC*mec* type IV, and the other as *t*595/ST152/SCC*mec* type V [22]. Additionally, *t*595/ST152/SCC*mec* type V was detected as nasal coloniser in patient and healthcare worker in a Serbian university hospital [23].

The first MRSA isolate with a previously unknown ST152 was recovered from a Danish patient in 2001 who was hospitalised in Kosovo (Serbia) [24]. This isolate exhibited *spa* type *t*207. PVL-positive ST152/SCC*mec* type V has been isolated sporadically throughout Central Europe, but most of these patients were connected to former Yugoslavian countries [25–27]. Characterising a number of MRSA PVL-positive isolates, Monecke et al. found one corresponding to ST152 and *spa* type *t*355 [28]. This strain was recovered from a child who emigrated from the North Macedonia. Based on this finding, Monecke et al. (2011) proposed that, in Europe, clonal lineage of ST152 emerged in Balkans and then dispersed to other regions [29].

In several studies authors noted a high prevalence of PVL-positive isolates of MSSA from African countries. The prevalence in Cameroon, Niger, and Senegal was 57%, being high both in hospital and community settings [30]. Moreover, the genetic diversity of PVL-positive MSSA isolates was high, and a significant proportion belonged to CC152 genetic backgrounds [31]. MLST analysis of nasal carriage isolates of *S*. *aureus* from hospitalised patients in Mali revealed that 23.9% of MSSA isolates, all PVL-positive, corresponded to ST152 [32]. Due to a high prevalence and diversity of PVL-positive ST152, Ruimy et al. (2008) speculated that this clone originated in Africa and has dispersed to Europe where it acquired methicillin resistance [32].

Similarly to ST152, *spa* type *t*355 is only sporadically found outside of Africa, and is almost always connected to ST152. *Spa* type *t*355 is detected in the Netherlands (3.4%), Sweden (2.8%) and in Croatia (82.6%) where it is the most prevalent *spa* type [33]. The total frequency of *t*355 is 0.33% (http://www.spatialepidemiology.net/srl-maps/maps). *T*1096 is another *spa* type noted to be connected to ST152 [34]. It is known that certain STs and *spa* types predominate in distinct geographic regions [33]. The reasons for persistence of *t*355/ST152 genotype for years in Serbia may include the adaptation of this organism to local host population and environmental conditions. Besides, the predominance of *t*355/ST152/SCC*mec* type V genotype in PVL-positive MRSA strains at MMA, although a few *spa* types are circulating in the community, may be a consequence of a clonal dispersion.

In our study, a single PVL-positive MRSA clone predominates. Similarly, one PVL-positive MRSA clone predominates in the United States (clone ST8-MRSA-IV/USA300), and in Australia (clone ST93-MRSA-IV) [35, 36]. Contrarily, in European countries PVL-positive MRSA isolates are generally polyclonal, with different clones being prevalent in different geographic regions, and temporal shifts occur in the predominant clonal type [37, 38]. In recent years the prevalent European CA-MRSA clone has been PVL-positive ST80/SCC*mec* type IV [39].

## Conclusion

A high prevalence of MRSA isolates and of CA-MRSA genotypes represents a grave health threat in the hospital. The detection of ST152/SCC*mec* type V genetic background in all PVL-positive isolates is of concern because of its high pathogenic potential. Our results highlight that *t*355/ST152/SCC*mec* type V is still the dominant PVL-positive clone in this region, with a propensity to replace other PVL-positive MRSA clones. To prevent further dispersion, it is not sufficient to implement preventive measures only in hospitals, but it has also to be done in the community.

## Supporting information

S1 Raw images Original, uncropped images of PCR and PFGE gels. (PDF)

S1 Table Raw data. Phenotypic and genotypic characteristics of *Staphylococcus aureus* strains analysed in the study.
(DOC)
