# Supplementary material for: Predominance of t355/ST152/SCCmec V clonal type among PVL-positive MRSA isolates in a tertiary care hospital in Belgrade, Serbia
Source: PLoS One. 2022 Sep 8;17(9):e0273474. doi: 10.1371/journal.pone.0273474 (PMC9455871; doi:10.1371/journal.pone.0273474)
Supplement: S1 Raw images — (PDF) [file pone.0273474.s001.pdf]

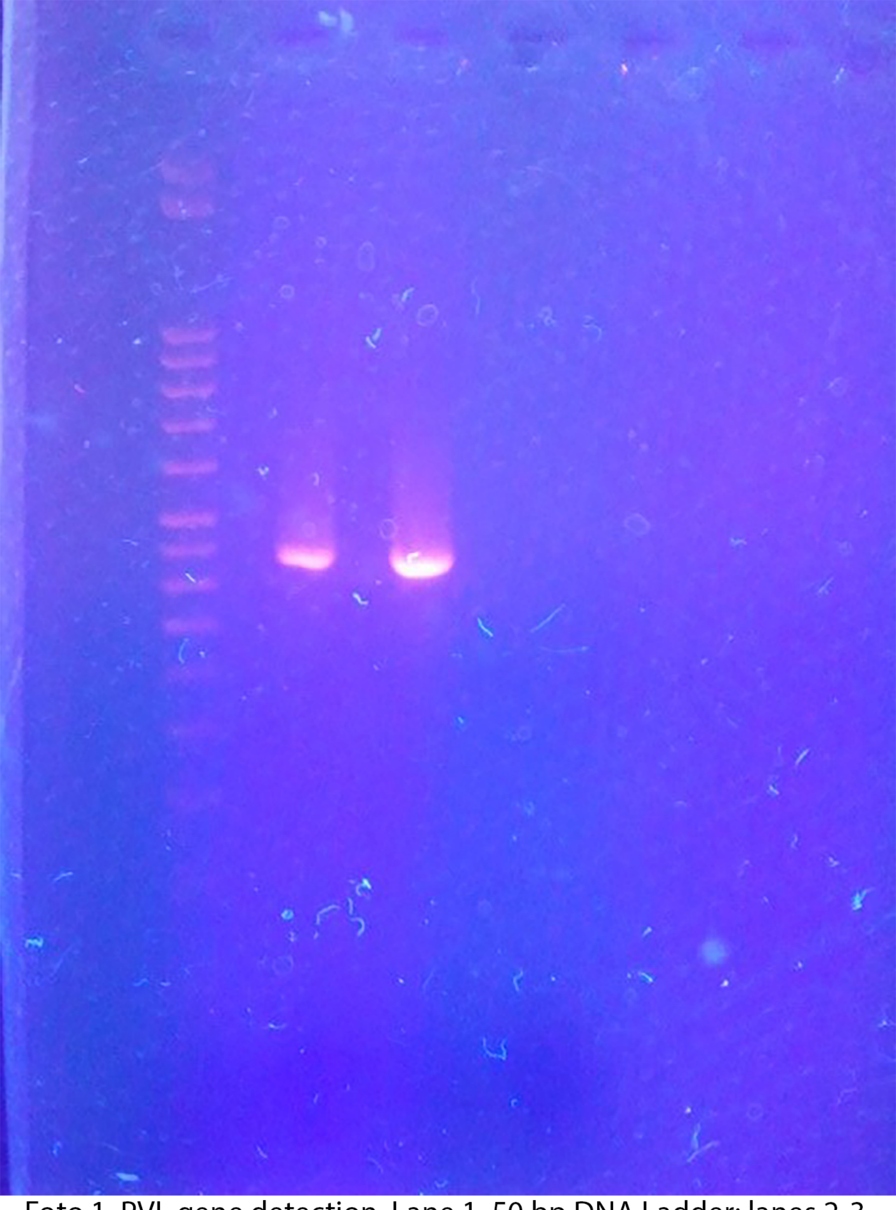

Foto 1. PVL gene detection. Lane 1, 50 bp DNA Ladder; lanes 2-3, MRSA clinical isolates 570 and 187; lane 4, negative PCR control.

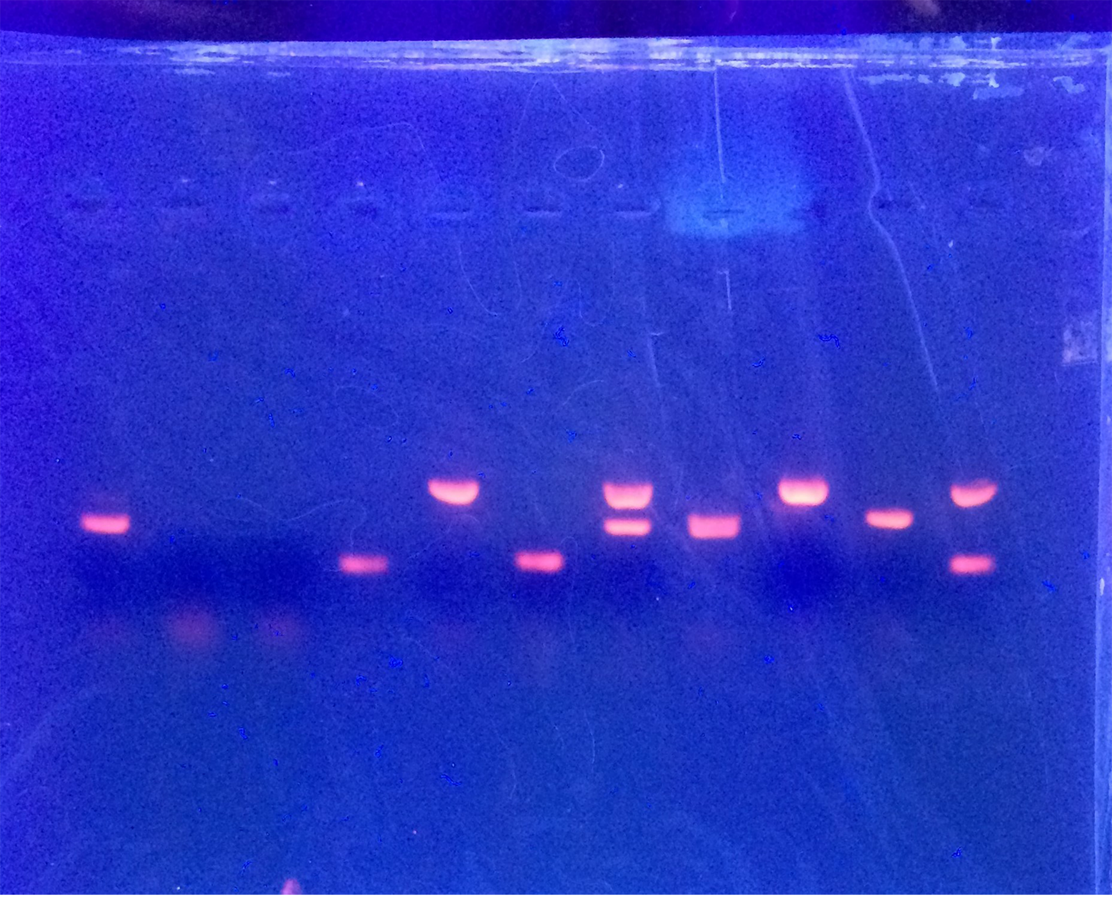

Foto 2. SCCmec typing of MRSA clinical isolates. Lanes 1-9, MRSA clinical isolates 384, 974, 983, 9051, 9087, 9501, 9676, 765, and 491; lane 10, standard strain HT20020290 containing type I SCCmec genetic element; lane 11, standard strain HT20030826 containing type III SCCmec genetic element.

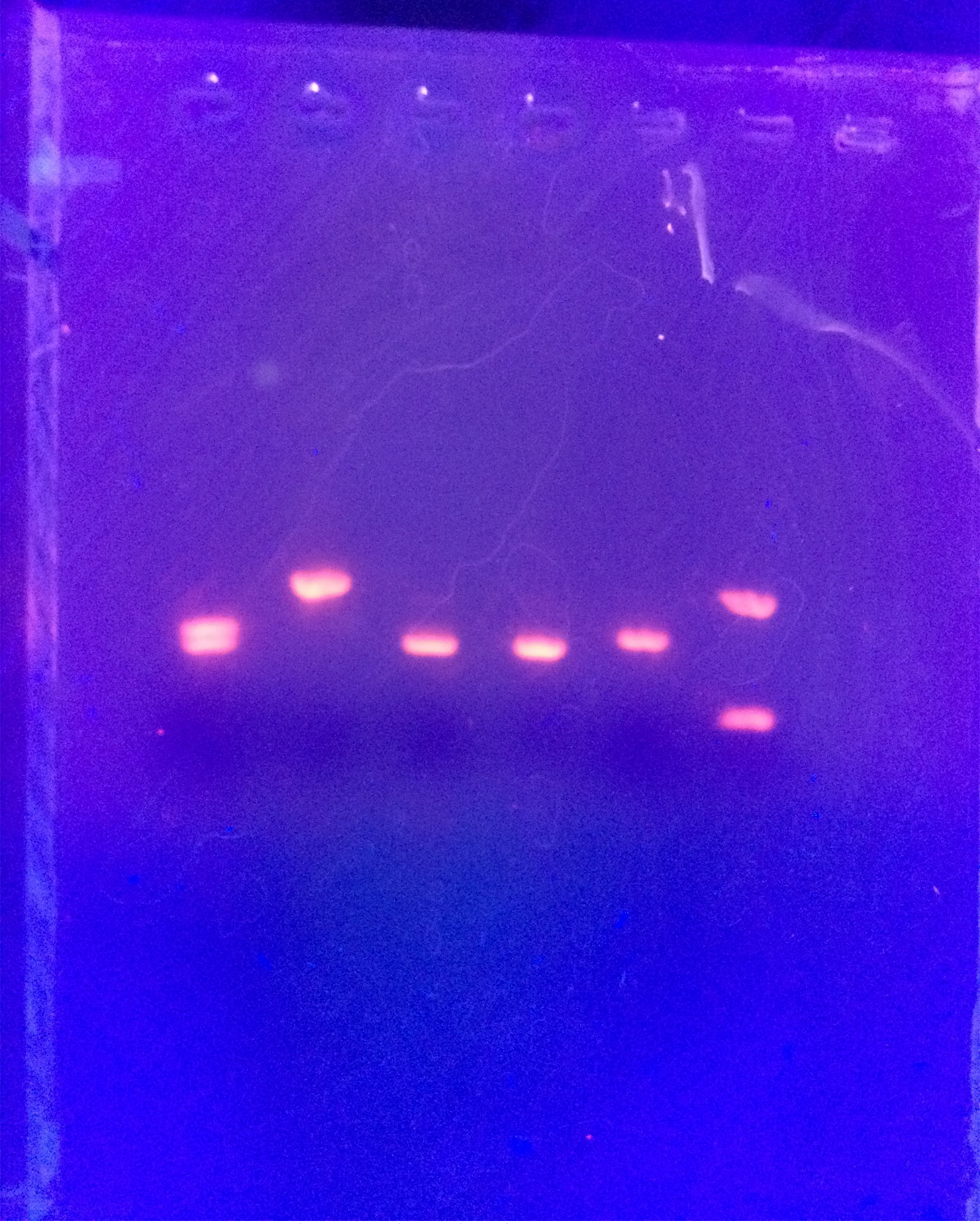

Foto 3. SCCmec typing of MRSA clinical isolates.
Lanes 1-4, MRSA clinical isolates 677, 700, 758, 882;
lane 5, standard strain HT20020290 containing type I SCCmec genetic element;
lane 6, standard strain HT20030826 containing type III SCCmec genetic element.

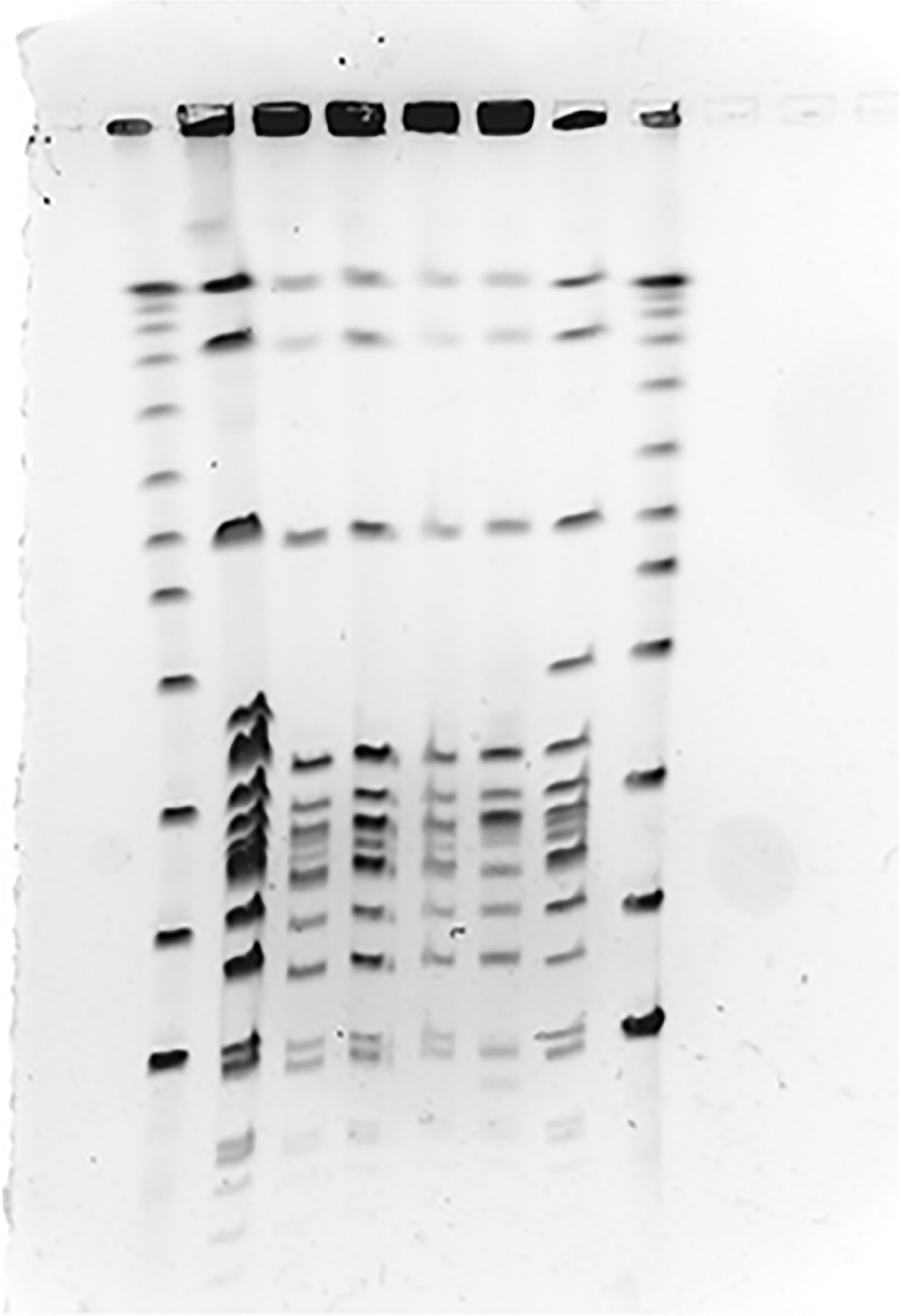

Figure . SmaI-PFGE macrorestriction profile of luk-PV-positive SCCmecV MRSA isolates. L, λ concatemers (New England Biolabs);
MRSA isolates: 87, 491, 544, 570, 4579, 4645.
