# Supplementary material for: Predominance of t355/ST152/SCCmec V clonal type among PVL-positive MRSA isolates in a tertiary care hospital in Belgrade, Serbia
Source: PLoS One. 2022 Sep 8;17(9):e0273474. doi: 10.1371/journal.pone.0273474 (PMC9455871; doi:10.1371/journal.pone.0273474)
Supplement: S1 Table — Phenotypic and genotypic characteristics of Staphylococcus aureus strains analysed in the study. (DOC) [file pone.0273474.s002.doc]

Table S1. Raw data. Phenotypic and genotypic characteristics of *Staphylococcus aureus* strains analysed in the study

|  | **Isolate Number** | ***mecA*** | ***pvl*** | **SCC*mec* type** | **Specimen** | **Antimicrobial Susceptibility** | | | | | | | | | |
| --- | --- | --- | --- | --- | --- | --- | --- | --- | --- | --- | --- | --- | --- | --- | --- |
|  |  |  |  |  |  | **Fox** | **Pen** | **Eritro** | **Klinda** | **S/T** | **Fus kis** | **Rif** | **Teico** | **Tetra** | **Levo** |
| 1. | **5073** | **+** | **-** | **-** | wound swab | + | + | + | + |  | + |  |  |  |  |
| 2. | **5479** | **+** | **-** | I | nasal swab | + | + | + | + |  |  |  |  | + |  |
| 3. | **5531** | **+** | **-** | III. | wound swab | + | + | + | + |  |  |  |  |  |  |
| 4. | **570/679** | **+** | **+** | V | wound swab | + | + |  |  |  |  |  |  |  |  |
| 5. | **5795** | **+** | **-** | IV | eye swab | + | + |  |  |  |  |  |  |  |  |
| 6. | **5796** | **+** | **-** | II | wound swab | + | + | + | + |  |  |  |  |  |  |
| 7. | **934/898** | **+** | **-** | III. | wound swab | + | + |  |  |  |  |  |  |  |  |
| 8. | **5806** | **+** | **-** | III. | aspirate | + | + | + | + | + | + | + |  |  | + |
| 9. | **5873** | **+** | **-** | III. | nasal swab | + | + | + | + |  |  |  |  |  |  |
| 10. | **5914** | **+** | **-** | III. | wound swab | + | + | + | + |  |  |  |  |  |  |
| 11. | **940/594** | **+** | **-** | IV | wound swab | + | + |  | + | + |  |  |  |  |  |
| 12. | **790** | **+** | **-** | V | urine |  |  |  |  |  |  |  |  |  |  |
| 13. | **1257** | **+** | **-** | III. | blood | + | + | + | + |  |  | + |  |  | + |
| 14. | **059 S** | **+** | **-** | III | nasal swab | + | + | + | + |  |  |  |  | + |  |
| 15. | **043** | **+** | **-** | III. | aspirate | + | + | + | + |  |  | + |  |  | + |
| 16. | **184** | **+** | **-** | IV | wound swab | + | + | + | + |  |  |  |  |  |  |
| 17. | **187** | **+** | **+** | V | wound swab | + | + | + | + |  |  |  |  |  |  |
| 18. | **068** | **+** | **-** | III. | wound swab | + | + | + | + |  |  | + |  |  | + |
| 19. | **371** | **+** | **-** | I | wound swab | + | + |  |  |  | + | + |  | + |  |
| 20. | **395** | **+** | **-** | I | wound swab | + | + | + | + |  | + |  |  |  |  |
| 21. | **211** | **+** | **-** | III. | wound swab | + | + | + | + |  |  | + |  |  | + |
| 22. | **845** | **+** | **-** | III | mouth swab | + | + | + | + |  |  | + |  | + | + |
| 23. | **6879** | **+** | **-** | I | nasal swab | + | + | + | + |  |  |  |  |  |  |

|  | **Isolate Number** | ***mecA*** | ***pvl*** | **SCC*mec* type** | **Specimen** | **Antimicrobial Susceptibility** | | | | | | | | | |
| --- | --- | --- | --- | --- | --- | --- | --- | --- | --- | --- | --- | --- | --- | --- | --- |
|  |  |  |  |  |  | **Fox** | **Pen** | **Eritro** | **Klinda** | **S/T** | **Fus kis** | **Rif** | **Teico** | **Tetra** | **Levo** |
| 24. | **212** | **+** | **-** | III. | wound swab | + | + |  |  |  |  |  |  |  |  |
| 25. | **309** | **+** | **-** | III. | wound swab | + | + | + | + |  |  |  |  |  |  |
| 26. | **1277** | **+** | **-** | III. | punctate | + | + | + | + |  |  | + |  |  | + |
| 27. | **422** | **+** | **-** | III. | wound swab | + | + |  |  |  |  |  |  |  |  |
| 28. | **438** | **+** | **-** | III. | wound swab | + | + |  |  |  |  |  |  |  |  |
| 29. | **7575** | **+** | **-** | I | nasal swab | + | + | + | + |  |  |  |  |  |  |
| 30. | **7577** | **+** | **-** | I | wound swab | + | + | + | + |  |  |  |  |  |  |
| 31. | **7585** | **+** | **-** | III. | wound swab | + | + | + |  |  |  |  |  |  |  |
| 32. | **7669** | **+** | **-** | I | wound swab | + | + | + | + |  |  |  |  |  |  |
| 33. | **7766** | **-** | **-** | - | wound swab | + | + |  |  |  | + |  |  |  |  |
| 34. | **7788** | **+** | **-** | I | wound swab | + | + |  |  |  |  |  |  |  |  |
| 35. | **932** | **+** | **-** | IV | nasal swab | + | + |  |  |  |  |  |  |  |  |
| 36. | **287** | **+** | **-** | III. | wound swab | + | + |  |  |  |  |  |  |  |  |
| 37. | **421** | **+** | **-** | III. | wound swab | + | + | + | + |  |  |  |  |  |  |
| 38. | **8481** | **+** | **-** | IV | wound swab | + | + |  |  |  |  |  |  |  |  |
| 39. | **476** | **+** | **-** | III. | sputum | + | + | + | + |  |  |  |  |  |  |
| 40. | **8544** | **+** | **-** | IV | nasal swab | + | + |  |  |  |  |  |  |  |  |
| 41. | **8483** | **+** | **-** | I | wound swab | + | + |  |  |  |  |  |  |  |  |
| 42. | **8572** | **+** | **+** | V | wound swab | + | + |  |  |  |  |  |  |  |  |
| 43. | **2027** | **+** | **-** | IV | blood | + | + |  |  |  |  |  |  |  | + |
| 44. | **1544** | **+** | **+** | V | wound swab | + | + | + | + |  |  |  |  |  |  |
| 45. | **121/9630** | **+** | **-** | V | nasal swab | + | + | + | + |  |  |  |  |  |  |
| 46. | **8851** | **+** | **-** | III. | wound swab | + | + | + | + |  |  |  |  |  | + |
| 47. | **8927** | **+** | **-** | III. | wound swab | + | + | + | + |  |  |  |  |  |  |
| 48. | **9316** | **+** | **-** | III. | wound swab | + | + | + | + |  |  | + |  |  | + |

|  | **Isolate Number** | ***mecA*** | ***pvl*** | **SCC*mec* type** | **Specimen** | **Antimicrobial Susceptibility** | | | | | | | | | |
| --- | --- | --- | --- | --- | --- | --- | --- | --- | --- | --- | --- | --- | --- | --- | --- |
|  |  |  |  |  |  | **Fox** | **Pen** | **Eritro** | **Klinda** | **S/T** | **Fus kis** | **Rif** | **Teico** | **Tetra** | **Levo** |
| 49. | **1660** | **+** | **-** | III. | tracheal swab | + | + |  |  |  |  |  |  |  | + |
| 50. | **9408** | **+** | **-** | III. | wound swab | + | + |  |  |  |  | + |  |  | + |
| 51. | **9435** | + | - | III | wound swab | + | + | + | + |  |  | + |  | + | + |
| 52. | **9592** | + | - | IV | wound swab | + | + |  |  |  |  |  |  |  |  |
| 53. | **65** | + | - | III. | punctate | + | + | + | + |  |  | + |  |  | + |
| 54. | **351** | + | - | III | wound swab | + | + | + | + |  |  | + |  | + | + |
| 55. | **559** | **+** | - | I | nasal swab | + | + | + | + |  |  |  |  |  |  |
| 56. | **1939** | + | - | I | nasal swab | + | + |  |  |  |  |  |  |  |  |
| 57. | **1927** | + | - | IV | nasal swab | + | + | + | + |  |  |  |  |  |  |
| 58. | **L 53** | + | - | IV | nasal swab |  |  |  |  |  |  |  |  |  |  |
| 59. | **2335** | + | - | IV | wound swab | + | + |  |  |  |  |  |  |  |  |
| 60. | **279/2382** | + | - | IV | nasal swab | + | + | + | + |  |  |  |  |  |  |
| 61. | **2429** | + | - | III. | wound swab | + | + |  |  |  | + |  |  |  |  |
| 62. | **2477** | + | - | IV | wound swab | + | + | + | + |  |  |  |  |  |  |
| 63. | **2943** | + | - | IV | wound swab | + | + |  |  |  |  |  |  |  |  |
| 64. | **4129** | + | - | V | nasal swab | + | + |  |  |  |  |  |  |  |  |
| 65. | **860** | + | - | III | aspirate | + | + |  |  |  |  | + |  |  | + |
| 66. | **4326** | + | - | III | wound swab | + | + | + | + |  |  |  |  |  |  |
| 67. | **440** | + | - | III | wound swab | + | + | + | + |  |  | + |  | + | + |
| 68. | **1154/480** | + | - | V | nasal swab | + | + |  |  |  |  |  |  | + |  |
| 69. | **4561** | + | - | III | Wound swab | + | + | + | + |  |  | + |  |  | + |
| 70. | **4568** | + | - | III | wound swab | + | + | + | + |  |  | + |  | + | + |
| 71. | **4579** | + | + | V | wound swab | + | + | + |  |  |  |  |  |  |  |
| 72. | **1349** | + | - | III | blood | + | + | + | + |  |  | + |  |  | + |
| 73. | **4645** | + | + | V | wound swab | + | + | + | + |  |  |  |  |  |  |

|  | **Isolate Number** | ***mecA*** | ***pvl*** | **SCC*mec* type** | **Specimen** | **Antimicrobial Susceptibility** | | | | | | | | | |
| --- | --- | --- | --- | --- | --- | --- | --- | --- | --- | --- | --- | --- | --- | --- | --- |
|  |  |  |  |  |  | **Fox** | **Pen** | **Eritro** | **Klinda** | **S/T** | **Fus kis** | **Rif** | **Teico** | **Tetra** | **Levo** |
| 74. | **4900** | + | - | III | wound swab | + | + |  |  |  |  |  |  |  | + |
| 75. | **4918** | + | - | III | wound swab | + | + | + | + |  |  |  |  | + | + |
| 76. | **980** | + | - | III | punctate | + | + | + | + |  |  | + |  |  | + |
| 77. | **990** | + | - | III | aspirate | + | + | + | + |  |  | + |  |  | + |
| 78. | **5082** | + | - | IV | wound swab | + | + |  |  | + |  |  |  |  |  |
| 79. | **5315** | + | - | II | punctate | + | + | + | + | + |  | + |  | + | + |
| 80. | **052** | + | - | II | wound swab | + | + | + | + |  | + |  |  |  | + |
| 81. | **372** | + | - | IV | wound swab |  | + | + |  | + | + |  |  |  |  |
| 82. | **5502** | + | - | V | wound swab | + | + |  | + | + |  |  |  | + | + |
| 83. | **5559** | + | - | IV | wound swab | + | + |  |  |  |  |  |  |  |  |
| 84. | **1812** | + | - | III | blood | + | + | + | + |  |  | + |  |  | + |
| 85. | **911** | + | - | V | urine | + | + |  |  |  |  |  |  |  |  |
| 86. | **846** | + | - | - | blood | + | + | + | + |  |  |  |  | + | + |
| 87. | **903** | + | - | - | nasal swab | + | + | + | + |  |  |  |  |  |  |
| 88. | **1516** | + | - | IV | nasal swab | + | + |  |  |  |  |  |  |  |  |
| 89. | **977/934** | + | - | IV | nasal swab | + | + |  |  |  |  |  |  |  |  |
| 90. | **180/1581** | + | - | IV | nasal swab | + | + |  |  |  |  |  |  |  |  |
| 91. | **222** | + | - | III | wound swab | + | + | + | + |  |  |  |  |  |  |
| 92. | **274** | + | - | III | wound swab | + | + |  |  |  |  | + |  |  | + |
| 93. | **365** | + | - | III | mouth swab | + | + |  |  |  |  | + |  | + |  |
| 94. | **995** | + | - | III | blood | + | + | + | + |  |  | + |  | + | + |
| 95. | **390** | + | - | III | wound swab | + | + |  |  |  |  | + |  |  |  |
| 96. | **418** | + | - | V | wound swab | + | + | + | + |  |  |  |  |  |  |
| 97. | **446/984** | **+** | **-** | IV | nasal swab | + | + |  |  |  |  |  |  |  |  |
| 98. | **468/1006** | **+** | **-** | - | nasal swab | + | + |  |  |  |  |  |  |  |  |

|  | **Isolate Number** | ***mecA*** | ***pvl*** | **SCC*mec* type** | **Specimen** | **Antimicrobial Susceptibility** | | | | | | | | | |
| --- | --- | --- | --- | --- | --- | --- | --- | --- | --- | --- | --- | --- | --- | --- | --- |
|  |  |  |  |  |  | **Fox** | **Pen** | **Eritro** | **Klinda** | **S/T** | **Fus kis** | **Rif** | **Teico** | **Tetra** | **Levo** |
| 99. | **6524** | **+** | **-** | III | wound swab | + | + | + | + |  |  |  |  |  |  |
| 100. | **1288** | **+** | **-** | III | aspirate | + | + | + | + |  |  | + |  | + | + |
| 101. | **6552** | **+** | **-** | I | nasal swab | + | + | + | + |  |  |  |  |  |  |
| 102. | **6627** | **+** | **-** | III | wound swab | + | + | + | + |  |  | + |  |  | + |
| 103. | **1327** | **+** | **-** | III | wound swab | + | + |  |  |  |  |  |  |  | + |
| 104. | **1365** | **+** | **-** | III | punctate | + | + | + | + |  |  | + |  |  | + |
| 105. | **5789** | **+** | **-** | I | throat swab | + | + | + | + |  |  |  |  |  |  |
| 106. | **2134** | **+** | **-** | III | blood | + | + |  |  |  |  | + |  |  | + |
| 107. | **2189** | **+** | **-** | III | blood | + | + | + |  |  |  | + |  |  | + |
| 108. | **6711** | **+** | **-** | III | wound swab | + | + |  |  |  |  | + |  |  | + |
| 109. | **6928** | **+** | **-** | IV | wound swab | + | + |  |  |  |  |  |  |  |  |
| 110. | **6947** | **+** | **-** | V | nasal swab | + | + |  |  |  |  |  |  |  |  |
| 111. | **055** | **-** | **-** | - | wound swab | + | + | + | + | + |  |  |  | + | + |
| 112. | **7205** | **+** | **-** | I | nasal swab | + | + | + | + |  |  |  |  |  |  |
| 113. | **7301** | **+** | **-** | I | nasal swab | + | + | + | + |  |  |  |  |  |  |
| 114. | **7293** | **+** | **-** | - | nasal swab | + | + |  |  |  |  |  |  |  |  |
| 115. | **7355** | **+** | **-** | III | wound swab | + | + |  |  |  |  | + |  |  | + |
| 116. | **1430** | **+** | **-** | - | aspirate | + | + |  |  |  |  | + |  |  | + |
| 117. | **1445** | **+** | **-** | - | wound swab | + | + | + | + |  |  | + |  |  | + |
| 118. | **2467** | **+** | **-** | III | blood | + | + | + |  |  |  | + |  |  | + |
| 119. | **7403** | **+** | **-** | III | wound swab | + | + | + | + |  |  | + |  |  |  |
| 120. | **627** | **+** | **-** | III | wound swab | + | + | + | + |  |  |  |  |  | + |
| 121. | **696** | **+** | **-** | V | nasal swab | + | + | + |  |  |  |  |  |  |  |
| 122. | **512** | **+** | **-** | IV | wound swab | + | + |  |  |  |  |  |  |  |  |
|  |  |  |  |  |  |  |  |  |  |  |  |  |  |  |  |
|  | **Isolate Number** | ***mecA*** | ***pvl*** | **SCC*mec* type** | **Specimen** | **Antimicrobial Susceptibility** | | | | | | | | | |
|  |  |  |  |  |  | **Fox** | **Pen** | **Eritro** | **Klinda** | **S/T** | **Fus kis** | **Rif** | **Teico** | **Tetra** | **Levo** |
| 123. | **7877** | + | - | III | wound swab | + | + | + | + |  |  | + |  |  | + |
| 124. | **7882** | + | - | III | wound swab | + | + |  |  |  |  | + |  |  | + |
| 125. | **7894** | + | - | III | wound swab | + | + | + | + |  |  | + |  |  | + |
| 126. | **7933** | + | - | III | wound swab | + | + | + | + |  |  | + |  |  | + |
| 127. | **8060** | + | - | III | punctate | + | + | + | + |  |  |  |  |  | + |
| 128. | **070** | + | - | I | eye swab | + | + | + |  |  |  |  |  |  |  |
| 129. | **1576** | + | - | III | CVC | + | + | + | + |  |  | + |  |  | + |
| 130. | **8131** | + | - | IV | wound swab | + | + | + |  |  |  |  |  |  |  |
| 131. | **2681** | + | - | III | blood | + | + | + |  |  |  |  |  |  |  |
| 132. | **2209** | + | - | V | nasal swab | + | + |  |  |  |  |  |  |  |  |
| 133. | **244** | + | - | III | wound swab | + | + | + | + |  |  | + |  |  | + |
| 134. | **2708** | + | - | IV | CVC | + | + |  |  |  |  | + |  |  | + |
| 135. | **8327** | + | - | III | nasal swab | + | + | + | + | + | + |  |  |  |  |
| 136. | **384** | + | - | V | nasal swab | + | + | + | + |  |  |  |  |  |  |
| 137. | **675** | + | - | I | wound swab | + | + | + | + |  |  |  |  |  |  |
| 138. | **8682** | + | - | IV | nasal swab | + | + |  |  |  |  |  |  |  |  |
| 139. | **8699** | + | - | III | wound swab | + | + |  |  |  |  | + |  |  |  |
| 140. | **686** | + | - | V | wound swab | + | + | + | + |  |  |  |  |  |  |
| 141. | **2871** | + | - | III | CVC | + | + | + | + |  |  | + |  | + | + |

SCC*mec*, staphylococcal chromosome cassette *mec*; *pvl*, Panton–Valentine leukocidin; Fox, cefoxitin; Pen, penicillin; Eritro, erythromycin; Klinda, clindamycin; S/T, trimethoprim-sulphametoxazole; Fus kis, fusidic acid; Rif, rifampin; Teico, teicoplanin; Tetra, tetracycline; Levo, levofloxacin
